# Reaction Behavior and Formation Mechanism of ZrB_2_ and ZrC from the Ni-Zr-B_4_C System during Self-Propagating High-Temperature Synthesis

**DOI:** 10.3390/ma16010354

**Published:** 2022-12-30

**Authors:** Jiaying Xu, Pengfei Ma, Binglin Zou, Xue Yang

**Affiliations:** 1 College of Science, Jilin Institute of Chemical Technology, Jilin 132022, China; 2 State Key Laboratory of Rare Earth Resources Utilization, Changchun Institute of Applied Chemistry, Chinese Academy of Sciences, Changchun 130022, China

**Keywords:** self-propagating high-temperature synthesis (SHS), ZrB_2_, ZrC, reaction behavior, formation mechanism

## Abstract

Self-propagating high-temperature synthesis (SHS) is a good way to prepare ZrB_2_-ZrC/metal cermet composites. In this work, ZrB_2_-ZrC/Ni cermet composites with various Ni contents were successfully fabricated by SHS using the Ni-Zr-B_4_C system. The effects of Ni content and particle size of the B_4_C powder on the SHS reaction were investigated. The results indicated that with an increase in Ni content, the adiabatic temperature, maximum combustion temperature, ignition delay time, and ceramic particle size in the product all showed a gradually decreasing trend. The SHS products and the ignition of the SHS reactions were significantly dependent on the B_4_C particle size. The formation mechanism of ZrB_2_ and ZrC during SHS from the Ni-Zr-B_4_C system was proposed based on the combustion wave quenching experiment.

## 1. Introduction

Particle reinforced metal matrix composites have attracted increasing attention because of their excellent performance. ZrB_2_ and ZrC have high hardness, a high melting point, good corrosion resistance, and excellent thermodynamic stability, and also exhibit outstanding compatibility with the metal matrix, making them ideal materials for particle reinforcement phases [1,2,3,4,5].

Self-propagating high temperature synthesis (SHS) is a good way to prepare particle reinforced metal matrix composites because of its numerous advantages, such as a rapid synthesis of materials, low energy consumption, and high product purity [6,7,8,9,10,11,12]. In recent years, it has been reported that ZrC-ZrB_2_/metal cermets were prepared by adding different metal elements into the Zr-B_4_C system and using metal-Zr-B_4_C as reaction system, and the reaction mechanism was investigated. Hu Qiaodan et al. [1] prepared ZrC-ZrB_2_/Al and studied the SHS reaction mechanism of the Al-Zr-B_4_C system, pointing out that Al plays a very important role in the Al-Zr-B_4_C system. At first, molten Al reacted with Zr, and then ZrAl_3_ formed the Al-Zr liquid phase, which provided a way for B and C atoms to enter the liquid phase, and finally, ZrC and ZrB_2_ precipitated out of the liquid. Zhang Mengxian et al. [4,5] studied the formation path of ZrB_2_ and ZrC in the Cu-Zr-B_4_C system during SHS using a differential scanning calorimeter (DSC) and X-ray diffraction (XRD). The effects of Cu content, B_4_C particle size, and heating rate on the SHS reaction behavior were also studied. Zhang Mengxian et al. [2,3] also studied the reaction behavior in the Co-Zr-B_4_C system during SHS. The ZrC-ZrB_2_ ceramic composite powders were in situ synthesized by SHS using the Co-Zr-B_4_C system, and then plasma was sprayed to form cermet coatings on an Mg alloy. The addition of metal can increase the contact area of the reactants by forming an intermediate liquid phase, thus reducing the difficulty of the reaction. Ni is a promising candidate, with a low wetting angle with the ceramic phases, and Ni can react with B_4_C to form an Ni-B liquid phase [7,9].

In our previous paper [13], ZrC-ZrB_2_/Ni cermet powders were successfully synthesized by SHS using an Ni-Zr-B_4_C system. The SHS-derived powders were deposited on an Mg alloy to form ZrC-ZrB_2_/Ni cermet coatings by using atmospheric plasma spraying. The produced coatings bonded well with the substrate and provided superior wear resistance. In another previous paper [14], the reaction mechanism in an Ni-Zr-B_4_C system to form ZrB_2_, and ZrC was analyzed by DSC and XRD. In general, DSC experimental conditions are slightly different from SHS reaction conditions in a glove box. Therefore, in this work, the combustion wave quenching experiment was used to reveal the SHS reaction mechanism in a glove box, and the effects of Ni content and different B_4_C reactants on the system products were studied. This is expected to provide a theoretical basis and guidance for the SHS of cermet composites.

## 2. Materials and Methods

The ZrB_2_–ZrC/Ni cermet composites were synthesized according to the following reaction equation:

(1) $$x{\mathrm{Ni}+3\mathrm{Zr}+B}_{4}C\to x\mathrm{Ni}+2{\mathrm{ZrB}}_{2}+\mathrm{ZrC}$$

**Figure 1:**
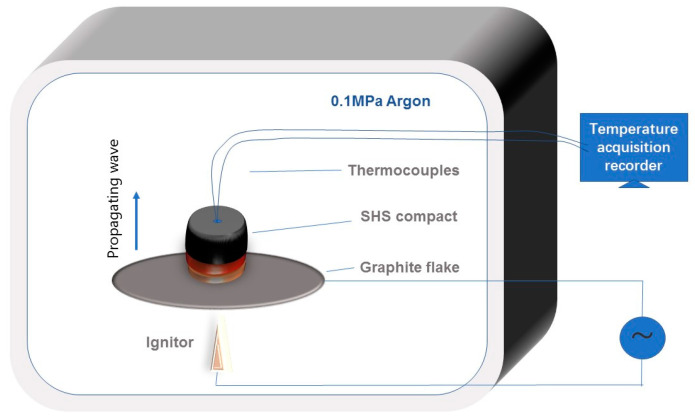
The schematic diagram of the SHS experimental apparatus.

Commercial Ni (≤48 μm, 99% purity, ST-nano science and technology Ltd. Co., Shanghai, China), Zr (≤38 μm, 99% purity, ST-nano science and technology Ltd. Co., Shanghai, China), and B_4_C (≤3.5 μm; ≤14 μm; ≤28 μm; ≤40 μm, ≤80 μm, 95% purity, Abrasive Ltd. Co., Dunhua, China) powders were used as the starting materials. When studying the influence of Ni content on the SHS reaction, the particle size of B_4_C was selected as 3.5 μm. The particle size of the B_4_C powders varied from 3.5 μm to 80 μm to investigate the effect of the reactant particle size. The Zr and B_4_C powders, with a ratio corresponding to that of stoichiometric 2ZrB_2_-ZrC (mole ratio) mixed with 0~50 wt.% Ni content, were selected for the powder blends. The raw reactant powders were dry-mixed by ball milling at a low speed (~50 rpm) for 6 h, and then pressed into cylindrical compacts (about 20 mm in diameter and 15 ± 2 mm in height) using a stainless steel die to acquire densities of 60 ± 2% theoretical density. The SHS reaction was performed in a self-made glove box filled with argon gas at 0.1 MPa. The green compact was placed on a thin graphite flake and subsequently ignited from the bottom by an arc welding flame with a strong current of 60 A. A small hole with a radius of 2 mm and a depth of 2 mm was drilled at the top of the compact. A pair of W–5% Re/W–26% Re thermocouples was inserted into the hole and linked up with an temperature acquisition recorder to obtain a time–temperature curve. The acquisition speed was 20 points per second. The schematic diagram of the SHS experimental apparatus is shown in Figure 1.

The phase composition of the SHS products was analyzed using an X-ray diffractometer (XRD) (D8 Advance, Bruker, Cu-Kα radiation, λ = 0.15406 nm, Germany) at a scanning rate of 6°/min and a scanning range of 20–80°. The microstructure of the SHS products was examined by scanning electron microscopy (SEM) (S-4800, Hitachi, Tokyo, Japan) equipped with energy dispersive spectroscopy (EDS). The linear intersection of the SEM image was used to measure the size of the ceramic particles.

**Figure 2:**
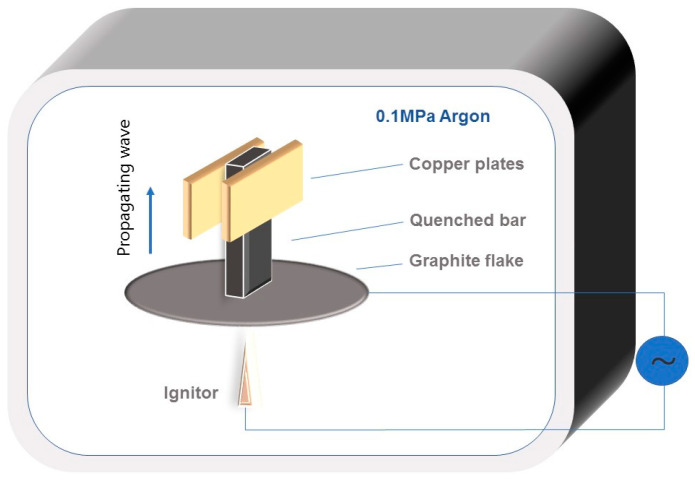
The schematic diagram of the combustion wave quenching experimental device.

The combustion wave quenching experiment is a good method to use for studying the reaction mechanism of SHS. The copper-mold-aided combustion wave quenching experiment, using the Ni-Zr-B_4_C system with 30 wt.% Ni in the compact, was performed. The particle size of B_4_C in the quenching experiment was 14 μm. Figure 2 shows the schematic diagram of the combustion wave quenching experimental device. When the combustion wave passed through a rectangular bar 65 mm × 10 mm × 5 mm in size, the heat loss increased due to the elongated shape of the bar and the cooling of two copper plates clamped in the middle of the bar, thus achieving the automatic flow blocking of the combustion wave. The quenched bar was carefully polished. The phase composition of the different regions of the SHS quenched bar was identified by X-ray micro-diffraction (D8 Discover with GADDS, Bruker AXS, Karlsruhe, Germany), which was operated at 40 kV and 30 mA using an 800 μm beam diameter. The microstructure of the Ni, Zr, B_4_C raw material powder and the different regions of the quenched bar were observed by SEM (S-4800, Hitachi, Japan), respectively. Element distribution at the combustion region was analyzed by EDS.

## 3. Results and Discussion

### 3.1. Reaction Behavior of the Ni-Zr-B_4_C System

#### 3.1.1. Effect of Ni Content on the SHS Reaction

The use of heat generated by the exothermic reaction itself for material synthesis is one of the most basic characteristics of SHS technology [8]. Therefore, thermodynamic analysis of the combustion system is the basis of studying the SHS process. The adiabatic temperature (*T*_ad_) is one of the most important thermodynamic parameters to describe the SHS reaction, which can be defined as the theoretically calculated temperature under an adiabatic condition during the SHS process. It can not only be used as a qualitative basis to judge whether the combustion reaction is self-propagating, but it can also predict the state of the combustion reaction products and provide a foundation for the composition design of the reaction system. Merzhanov et al. [15] proposed an empirical criterion such that when *T*_ad_ ≥ 1800 K, the SHS reaction can be self-propagating. *T*_ad_ can be calculated by computer programming using thermodynamic data from Ref. [16], according to Equation (2), as follows [15]

(2) $$\Delta H\left(298\right)+\int_{298}^{T_{ad}\left(298\right)}\sum^{\;}n_{j}C_{p}\left(P_{j}\right)dT+\sum_{298-T_{ad}\left(298\right)}n_{j}L\left(P_{j}\right)=0$$

where Δ*H* (298) is the reaction enthalpy at 298 K, *C_p_*(*P_j_*) and *L*(*P_j_*) are the heat capacity and latent heat of the products (if a phase change takes place), and *P_j_* and *n_j_* refer to the products and the stoichiometric constant, respectively.

**Figure 3:**
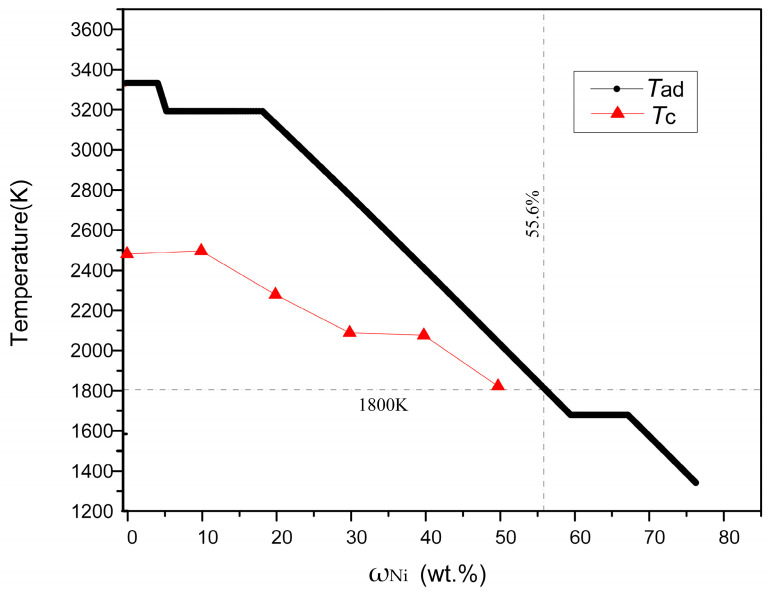
Variation in the *T*_ad_ and *T*_c_ of the Ni–Zr–B_4_C system with various Ni contents.

The variation in *T*_ad_ with Ni content (ω_Ni_) is shown in Figure 3. When ω_Ni_ is between 0–4 wt.%, 5.23–17.66 wt.% and 57.62–65.04 wt.%, respectively, three temperature platforms appear in the figure. The temperatures are 3323 K, 3187 K, and 1726 K, corresponding to the melting point of ZrB_2_, the boiling point of Ni, and the melting point of Ni, respectively. In these three platform ranges, the *T*_ad_ remains constant as the Ni content changes. This is because materials need to absorb a certain amount of heat during phase changes such as melting and gasification. Outside the three platforms, the *T*_ad_ decreases with the increase in Ni content. This is due to the increase in Ni content, which leads to a decrease in the amount of Zr and B_4_C, and a decrease in the heat released from the reaction. As shown in Figure 3, when the ω_Ni_ is 55.6 wt.%, the calculated *T*_ad_ is 1800 K. According to the empirical criterion, when *T*_ad_ ≥ 1800 K, the combustion reaction can be self-sustained [15]. Therefore, the range of 0% ≤ ω_Ni_ ≤ 50% was selected in this work. The SHS reactions of the Ni-Zr-B_4_C system with ω_Ni_ = 0, 10, 20, 30, 40, and 50 wt.% were all successfully ignited and self-propagated, which was consistent with the theoretical prediction.

**Figure 4:**
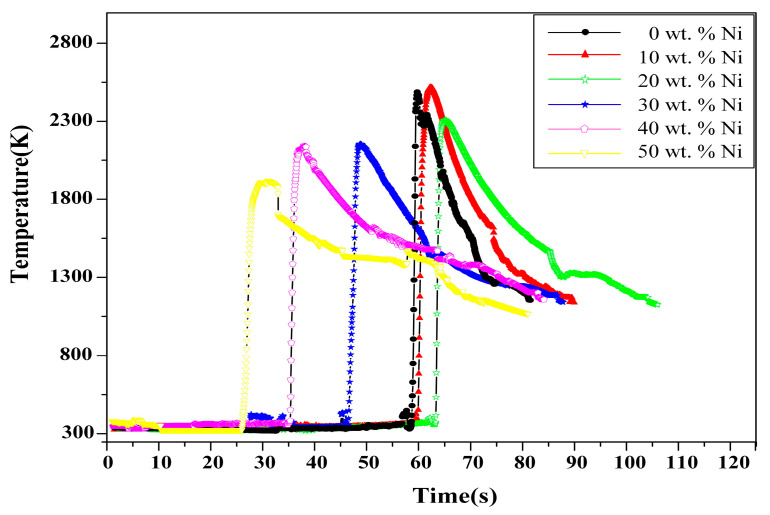
Time–temperature curves of the reactant compacts with various Ni contents during the SHS process.

Figure 4 shows the SHS combustion time–temperature curves of the reactant compacts with various Ni contents in the Ni-Zr-B_4_C system. According to the curves, the maximum combustion temperature (*T*_c_) decreases with the increase in Ni content. The *T*_c_ of each content was plotted as a curve and compared with *T*_ad_, as observed in Figure 3. It is revealed that the value of *T*_c_ is smaller than that of the corresponding *T*_ad_ due to heat loss and incomplete conversion in the actual SHS experiment [3,9]. Moreover, with the increase in Ni content, the difference between *T*_c_ and *T*_ad_ decreases gradually. It is worth mentioning that the type of time–temperature curve (yellow curve) changes when 50% Ni is added. At the peak of the curve, the temperature drops more slowly, and temperature peak smoothing is observed. It is presumed that in this case, the exothermic reactions of the formation of the final product are less intense than in other systems [17,18,19]. With the increase in Ni content, the heat release decreases gradually.

**Figure 5:**
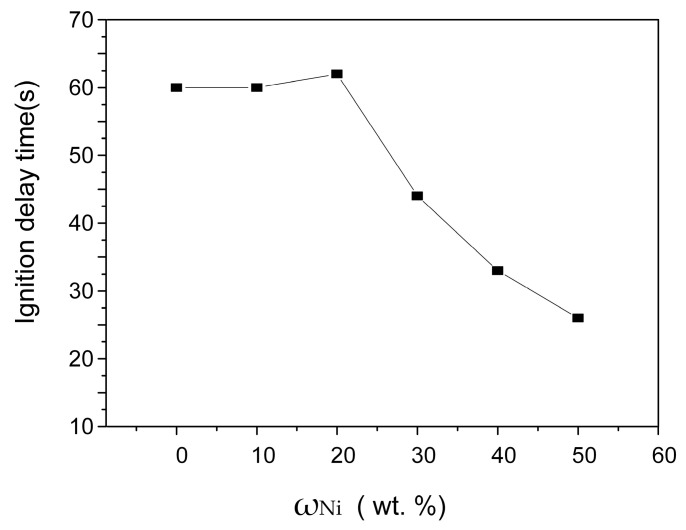
Variation in the ignition delay time (*t*_ig_) with various Ni contents.

The influence of Ni content on the SHS reaction ignition delay (*t*_ig_) can also be obtained from the combustion temperature measurement results of samples with different Ni content, as illustrated in Figure 5. With the Ni content increasing, *t*_ig_ shows a decreasing trend. Therefore, adding an appropriate amount of Ni to the Zr-B_4_C system can promote the ignition reaction. Previous investigations [14] have studied the formation path of ZrB_2_ and ZrC ceramic particles in the Ni-Zr-B_4_C system under DSC conditions, pointing out that initially, Ni reacts with B_4_C and Zr, which can form Ni-B and Ni-Zr melt in the subsequent heating process. The liquid melt is tightly wrapped with B_4_C. It provides a convenient way for B and C atoms to dissolve into the liquid phase, so that the Ni-Zr-B-C quaternary liquid phase can be easily formed, which accelerates the reaction precipitation of ZrB_2_ and ZrC. Therefore, the addition of Ni makes the SHS reaction easier to ignite [3].

**Figure 6:**
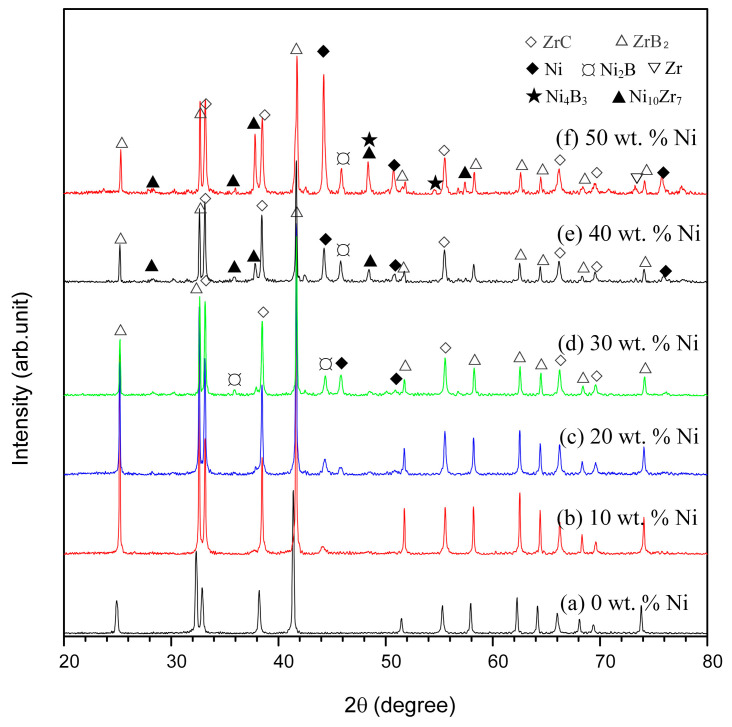
XRD patterns for the SHS products with different reactant Ni contents in Ni-Zr-B_4_C system: (a) 0 wt.%, (b) 10 wt.%, (c) 20 wt.%, (d) 30 wt.%, (e) 40 wt.%, and (f) 50 wt.%.

Figure 6 shows the XRD patterns for the products in the Ni-Zr-B_4_C system with different reactant Ni contents. The phase composition of the SHS products with 0 wt.% Ni consists of ZrB_2_ and ZrC. When ω_Ni_ = 10–30 wt.%, the combustion synthesis products contain a small amount of Ni_2_B in addition to Ni, ZrB_2_, and ZrC. When ω_Ni_ = 40–50 wt.%, the intermediate phases Ni_10_Zr_7_ and Ni_4_B_3_ appear and increase with the increase in Ni content, while Ni_2_B decrease with the increase in Ni content. This indicates that when the Ni content is greater than 40 wt.%, the incomplete degree of SHS reaction gradually increases, and too much Ni blocks the reaction of Zr and B_4_C. In addition, it can also be noted in the figure that when the Ni content is low, the peak intensity of ZrB_2_ is higher than that of ZrC, but with the increase in Ni content, the peak intensity of ZrB_2_ gradually becomes weaker than that of ZrC. This occurs because with the increase in Ni content, the reaction heat release of the system decreases, and the degree of incomplete reaction increases. A large amount of intermediate Ni_2_B has not yet participated in the reaction to form ZrB_2_.

**Figure 7:**
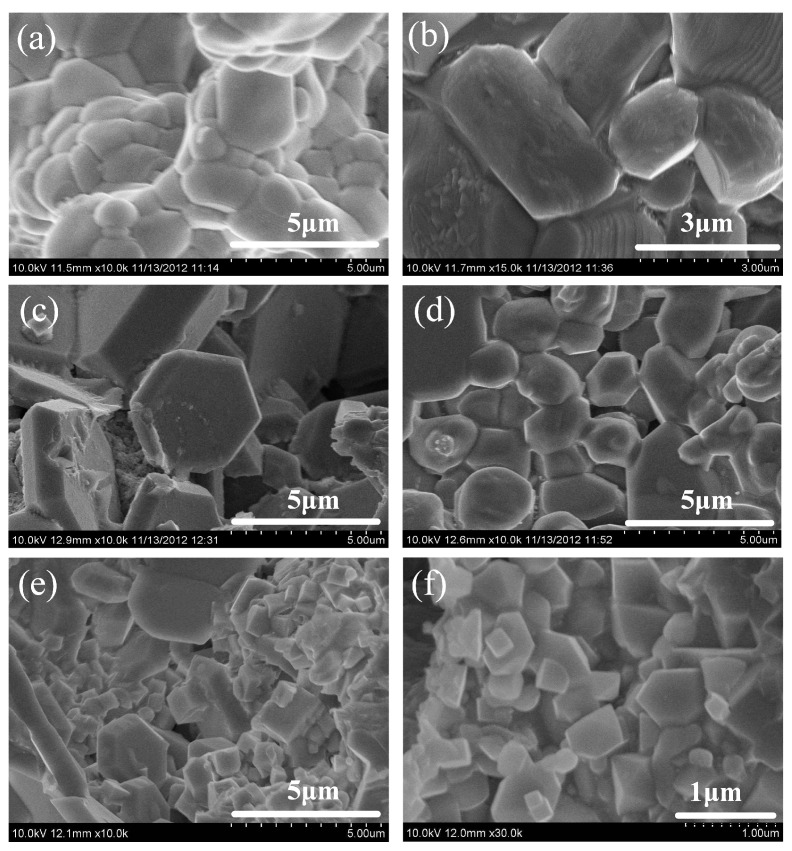
Microstructures of the SHS products with the reactant Ni contents of (**a**) 0 wt.%, (**b**) 10 wt.%, (**c**) 20 wt.%, (**d**) 30 wt.%, (**e**) 40 wt.%, and (**f**) 50 wt.%.

Figure 7 exhibits the microstructures of the SHS products with different Ni contents. It can be seen that granular ZrB_2_ and ZrC are formed in the product. The flat hexagonal particles are ZrB_2_, and the cube particles are ZrC. When the Ni content is 0 wt.%, the ceramic particles show severe sintering, and a large number of obvious holes can be observed in the low magnification photos. When the Ni content increases from 10 wt.% to 50 wt.%, the ceramic particle size gradually decreases from ~5 μm to ~0.5 μm. The main reasons for the decrease in ceramic particle size may be as follows: (1) the crystal growth is an exponential function of temperature [20]. With the increase in Ni content, the combustion temperature gradually decreases, and the growth rate of ZrB_2_ and ZrC decreases; (2) with the increase in Ni content, the thermal conductivity of the product increases, making the cooling rate increase, which is not conducive to the growth of ceramic particles [21]; and (3) with the increase in Ni content, the liquid phase between the ceramic particles increases during the reaction process, which hinders the sintering growth between the grains and reduces the trend of grain coarsening.

#### 3.1.2. Effect of B_4_C Particle Sizes on the SHS Reaction

The 30 wt.% Ni-Zr-B_4_C system was used as the research object. In the reaction mixture, the particle size of B_4_C was 3.5 μm, 14 μm, 28 μm, 40 μm, and 80 μm, the particle size of Ni was 48 μm, and the particle size of Zr was 38 μm.

**Figure 8:**
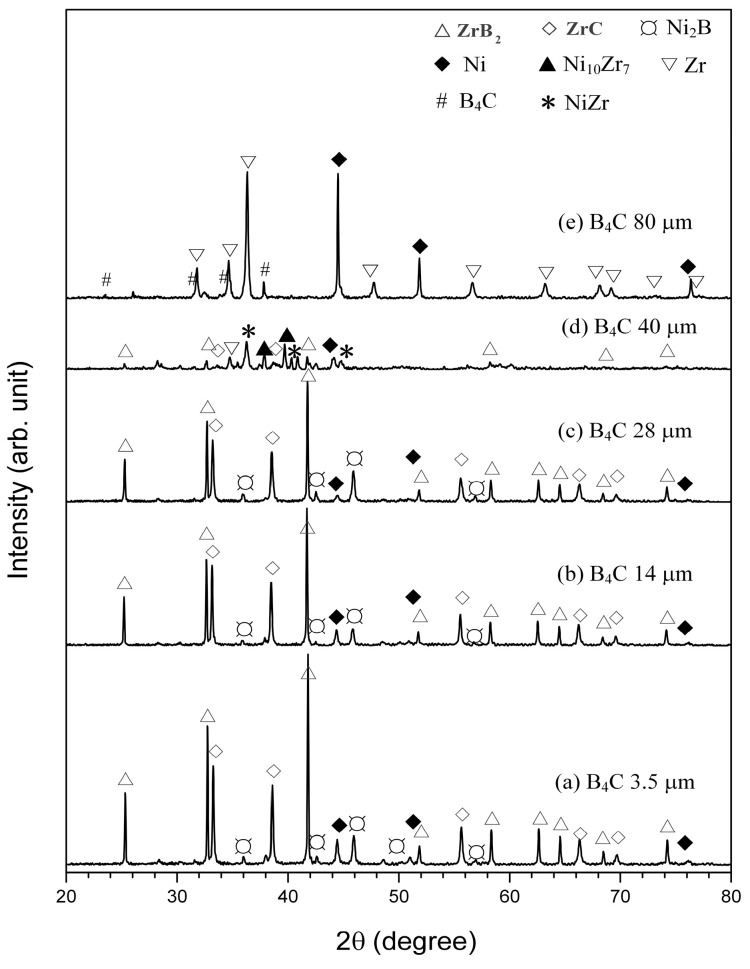
XRD patterns for SHS products of the 30 wt.% Ni-Zr-B_4_C system with various B_4_C particle sizes: (a) 3.5 μm, (b) 14 μm, (c) 28 μm, (d) 40 μm, and (e) 80 μm.

The products after the reaction of the samples with different B_4_C powder were analyzed by XRD, as illustrated in Figure 8. When the particle size of the B_4_C powder in the reactants is 3.5 μm, 14 μm, and 28 μm, the SHS products were composed of ZrB_2_, ZrC, Ni, and a small amount of intermediate Ni_2_B. With the increase in the particle size of the B_4_C powder, the ignition delay time of the SHS reaction increased. When the B_4_C size in the reactant was larger than 40 μm, the self-propagating reaction became very difficult, and the ignition time was longer. The product contained large amounts of NiZr, Ni_10_Zr_7_, and Ni_2_B, but the amounts of ZrB_2_ and ZrC were very small. When the particle size of the B_4_C powder in the reactant was 80 μm, the reaction could not be self-propagated, and almost no ZrB_2_ and ZrC were generated. The above results show that the SHS reaction behavior and the products of the 30 wt.% Ni-Zr-B_4_C system were significantly affected by the size of the B_4_C particles. The increase in the B_4_C particle size makes the ignition and propagation process of the self-propagating reaction difficult, and also reduces the propagation rate of the combustion wave and the product formation rate. A similar effect was found in the Cu-Zr-B_4_C system, in which coarser B_4_C particles postponed the formation of ZrB_2_ and ZrC [5]. The results show that the dissolution rate of B_4_C in Cu-Zr liquid decreased with the increase in B_4_C size, which could retard the formation of the Cu-Zr-B-C liquid. This led to the incomplete conversion of ZrB_2_ and ZrC.

### 3.2. Formation Mechanism of ZrB_2_ and ZrC during the SHS Process

#### 3.2.1. DSC Analysis

The reaction mechanism of the 30 wt.% Ni-Zr-B_4_C system during the DSC experiment was described in detail in a previous paper [14]. It was proposed as follows: firstly, Ni, B_4_C, and Zr have solid-state diffusion reactions to form some Ni_x_Zr_y_ and Ni_x_B_y_ intermetallics. Then, an Ni-B eutectic liquid formed at about 1025 °C, and the free C atoms dissolved into the Ni-B liquid to form an Ni-B-C ternary liquid. When the mixture was heated to about 1088 °C, part of Zr powder directly reacted with B_4_C through solid-state diffusion reaction, and part of the Zr powder dissolved into the Ni-B-C ternary liquid to form the Ni-Zr-B-C quaternary liquid. When the temperature reached 1150 °C, an Ni-Zr eutectic liquid formed. The Ni-Zr eutectic liquid could also dissolve into the Ni-B liquid or Ni-B-C liquid to form Ni-Zr-B-C quaternary liquid. Finally, ZrB_2_ and ZrC precipitated out of the saturated liquid.

#### 3.2.2. Combustion Wave Quenching Experiment

Although the above DSC analysis result is very helpful to understand the formation mechanism of the Ni-Zr-B_4_C system, the DSC experimental condition is different from the SHS in the glove box in terms of heating rate, sample volume, and compacting rate. These factors have a great influence on the reaction kinetics and mechanism of the system. Therefore, the reaction mechanism under the DSC condition cannot be used to fully explain the reaction mechanism under the SHS mode. In order to study the reaction mechanism of the ZrB_2_ and ZrC of the Ni-Zr-B_4_C system formed by the SHS in glove box, a combustion wave quenching experiment was conducted. The quenched sample was analyzed by XRD and SEM, and the reaction mechanism was studied.

**Figure 9:**
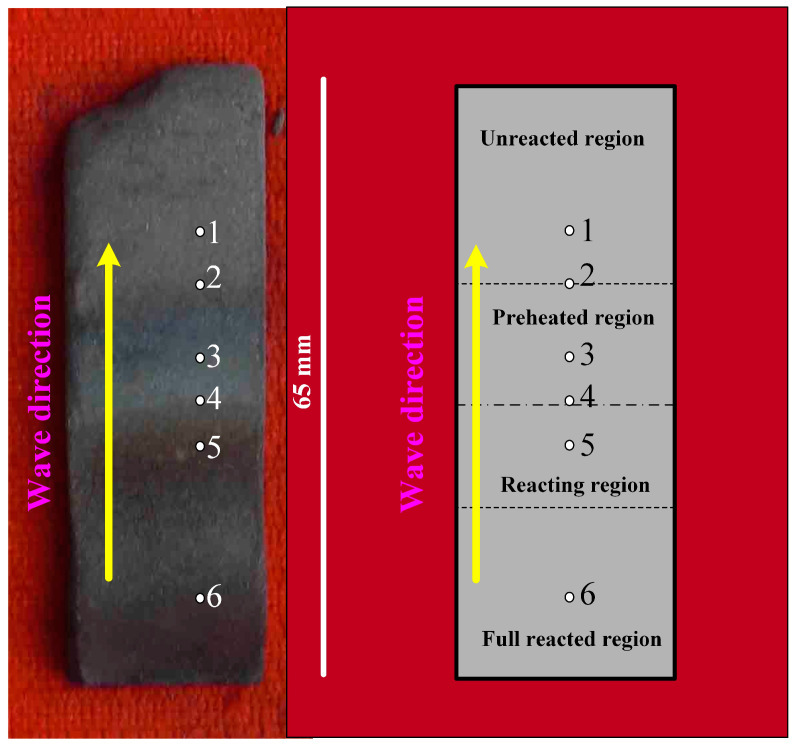
Macroscopic morphology and partition diagram showing different regions of the SHS quenched bar.

**Figure 10:**
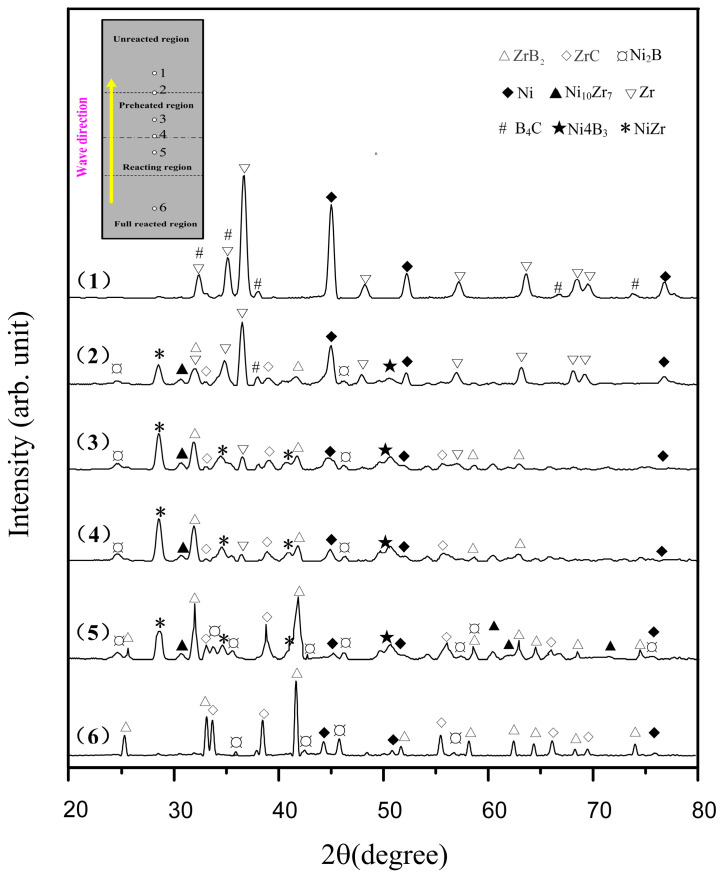
X-ray micro-diffraction patterns in different regions of the SHS quenched bar: (1) unreacted region, (2) preheated region, (3–5) reacting region, and (6) fully reacted region, respectively.

Figure 9 shows the macroscopic morphology and partition diagram of the SHS quenched bar, in which the wavy area with the darkest color is the typical morphology of the combustion wave. Against the spreading direction of combustion wave, the quenched bar can be differentiated into four regions according to the degree of reaction, namely, the unreacted region, the preheated region, the reacting region, and the fully reacted region. As shown in Figure 9, six points are noted in each reaction region and the interface between the two regions, respectively. Figure 10 shows the X-ray micro-diffraction patterns for each point.

**Figure 11:**
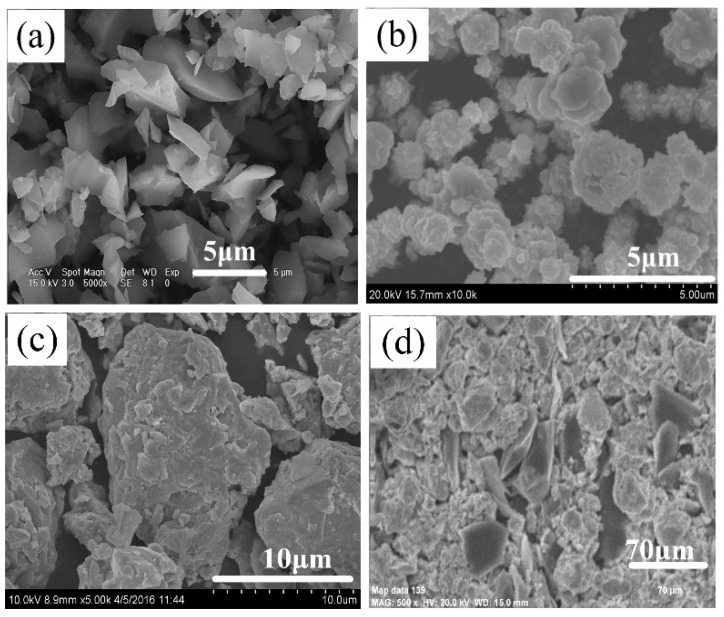
Microstructure of (**a**) Ni, (**b**) Zr, (**c**) B_4_C raw material powder, and (**d**) the unreacted region of the quenched bar.

**Figure 12:**
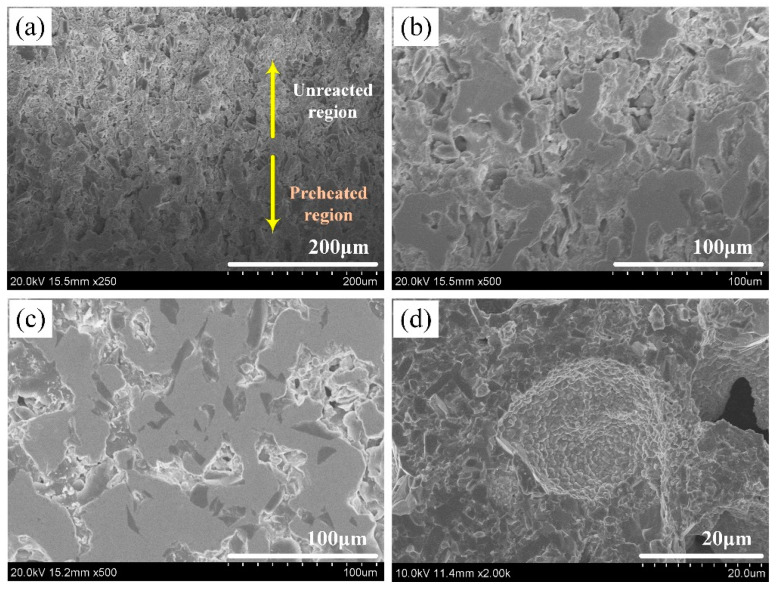
Microstructure of the (**a**) interface between the unreacted region and the preheated region, (**b**) preheated region, (**c**) reacting region, (**d**) fully reacted region, respectively.

Figure 11 shows the microstructure of the Ni, Zr, B_4_C raw material powder and the unreacted region of the quenched bar. It is observed that the Ni, Zr, and B_4_C powders can be easily distinguished from the morphology. Among them, the Ni particles show clusters of flowers, the Zr particles show smooth clumps, and the B_4_C particles show irregular shapes with sharp corners (see Figure 11a–c). In addition, the distribution of the reactants in the unreacted region is relatively uniform (see Figure 11d). The interface between the unreacted region and the preheated region and the typical morphology of the preheated region are presented in Figure 12a,b, respectively. It can be observed from Figure 12a that the morphology of the unreacted region is obviously different from that of the preheated region. The unreacted region is composed of a loose reactant powder mixture, while the preheated region is relatively dense, and there is an interface region between them. The change from point (2) to point (3) in the XRD results is shown in Figure 10, indicating that Ni reacted with Zr in the preheated region, forming NiZr, with a high content. Meanwhile, the formation of Ni_2_B and Ni_4_B_3_ indicated that a solid diffusion reaction also occurred between Ni and B_4_C. With the increase in temperature, Ni_2_B and Ni_4_B_3_ could form the Ni-B liquid phase when they reached the eutectic point (1291 K) [22], which rapidly spread out and filled into the pores of the sample, thus forming a relatively dense structure, as shown in Figure 12b.

It is worth mentioned that some papers [22] stated that TiC, rather than TiB_2_, would preferentially form in the metal-Ti-B_4_C system. However, the outcomes of this work are different, and there is no preferential formation of ZrC in the preheated region because the eutectic temperature of Ni_x_Zr_y_ is much larger than that of the reported Ni_x_Ti_y_.

Figure 12c exhibits the typical morphology of the reacting region. This region was more compact than the preheated region due to more liquid phase filling. It could also be observed that B_4_C was tightly surrounded by the liquid phase, and a portion of B_4_C had reacted. According to X-ray micro-diffraction and previous DSC results, the reaction between Zr and B_4_C occurred first in this region, and a great quantity of exothermic heat was released. The temperature of the system increased to reach the eutectic temperature of Ni_10_Zr_7_-Ni (1423 K) and NiZr-Ni (1443 K) [23], and the Ni-Zr liquid phase was formed. The quaternary Ni-Zr-B-C liquid phase was formed after dissolving with the Ni-B liquid phase and dissolving some C atoms; then, a great quantity of ZrB_2_ and ZrC were precipitated from the liquid phase.

**Figure 13:**
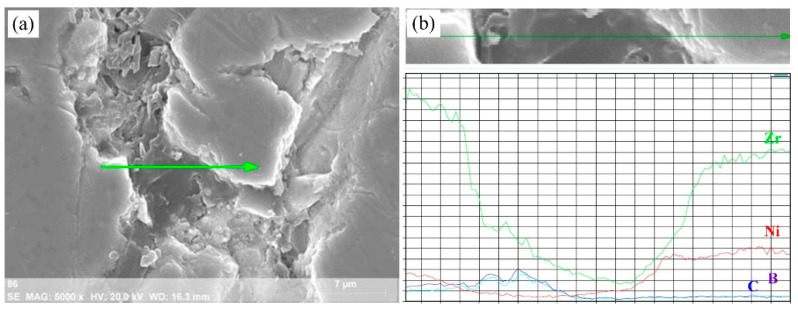
Microstructure of a remnant B_4_C particle dissolving into the Ni–Zr–B–C melt and the corresponding EDS-line analysis in the combustion region: (a) SEM image and (b) EDS-line analysis.

Figure 13 shows the morphology around the B_4_C particles in the reacting region and the EDS-line analysis of each element. The left part of the figure shows the interface between Zr and B_4_C, and a high content of the Zr element was also detected near the interior of B_4_C, indicating that a solid–solid reaction between Zr and B_4_C occurred, and a portion of ZrB_2_ and ZrC were formed through this reaction. The right part of the figure shows the interface between the Ni-Zr liquid phase and B_4_C, where parts B and C could obviously diffuse into the Ni-Zr liquid phase.

The microstructure of the fully reacted region is illustrated in Figure 12d. With the formation and saturation of a great quantity of the Ni-Zr-B-C liquid phase, a large amount of ZrB_2_ and ZrC was precipitated. It can be seen that the B_4_C particles were decomposed, and some holes were left at the original positions of the B_4_C particles. 

Therefore, the reaction mechanism in the Ni-Zr-B_4_C system during SHS is proposed as follows: (1) Ni + Zr + B_4_C → (2) NiZr + Ni_10_Zr_7_ + Ni_2_B + Ni_4_B_3_ + Ni + Zr + B_4_C → (3) NiZr + Ni_10_Zr_7_ + Ni-B (liquid) + ZrB_2_ + ZrC → (4) Ni-Zr (liquid) +Ni-B (liquid) or Ni-B-C (liquid) + ZrB_2_ + ZrC → (5) Ni-Zr-B-C (liquid) + ZrB_2_ + ZrC → (6) ZrB_2_ + ZrC + Ni.

Based on the results of the DSC analysis and quenching experiment, it was determined that the reaction mechanism of ZrB_2_ and ZrC under the two conditions was basically the same. Initially, Ni reacted with B_4_C and Zr to form Ni_2_B, Ni_4_B_3_, NiZr, Ni_10_Zr_7_, and other intermediate phases. As the temperature increased, Ni_2_B and Ni_4_B_3_ formed an Ni-B eutectic liquid phase. When the temperature increased further, some Zr directly reacted with B_4_C, and a large amount of heat was released to promote the temperature increase in the system. After reaching the eutectic temperature of Ni_10_Zr_7_, NiZr, and Ni, an Ni-Zr binary liquid phase was formed. When the two binary liquids mixed with each other, and some free C dissolved into it, the Ni-Zr-B-C quaternary liquid phase was formed. Finally, when the concentration of [Zr], [B], and [C] in the liquid met the conditions for the formation of ZrB_2_ and ZrC, a large amount of ZrB_2_ and ZrC precipitated out of the saturated liquid.

The difference is that under the DSC condition, the reactant system showed a loose morphology, a small heating rate, and a large heat loss, leading to a slow liquid phase formation rate, and a long reaction time to precipitate ZrB_2_ and ZrC from the liquid phase. In the SHS reaction, the reactant had a high heating rate and a small heat loss, which can quickly form the liquid phase and instantly generate a large amount of ZrB_2_ and ZrC.

## 4. Conclusions

ZrB_2_-ZrC/Ni cermets were successfully synthesized by SHS using the Ni-Zr-B_4_C system. The SHS reaction behavior and the formation mechanism of ceramic particles were systematically studied.

(1) With the increase in Ni content, the adiabatic temperature (*T*_ad_), the maximum combustion temperature (*T*_c_), the ignition delay time (*t*_ig_), and the ceramic particle size in the product all showed a gradually decreasing trend. When the content of Ni was low, the product was mainly composed of Ni, ZrB_2_, and ZrC. When the content of Ni exceeded 40 wt.%, a large number of the intermediate phases existed in the product.

(2) With the increase in B_4_C powder size, the ignition and propagating process of the SHS reaction became more and more difficult, and the spread rate of combustion wave and the formation rate of product gradually decreased. When the particle size of the B_4_C powder was larger than 40 μm, the product contained a large number of intermediate phases.

(3) It is revealed that the formation mechanism of ZrB_2_ and ZrC in the Ni-Zr-B_4_C system under the DSC condition and the SHS reaction in the glove box is basically the same. Initially, Ni reacted with B_4_C and Zr to form some intermediates such as Ni_2_B, Ni_4_B_3_, NiZr, and Ni_10_Zr_7_, and then the Ni-B eutectic liquid phase formed. As a part of Zr directly reacted with B_4_C, a great quantity of heat was released to promote the increase in the system temperature, and the Ni-Zr binary liquid phase formed. When the two binary liquids mixed with each other and some free C dissolved into it, the Ni-Zr-B-C quaternary liquid phase formed. Finally, a great quantity of ZrB_2_ and ZrC were precipitated out of the saturated liquid. These results are expected to provide a theoretical basis for the formation mechanism of ZrB_2_-ZrC/metal cermet composites using the SHS method.

## Data Availability

Not applicable.

## References

[1] Hu Q.D., Luo P., Zhang M.X., Song M.S., Li J.G. (2012). Combustion and formation behavior of hybrid ZrB_2_ and ZrC particles in Al-Zr-B_4_C system during self-propagation high temperature synthesis. Int. J. Refract. Met. Hard Mater..

[2] Zhang M.X., Huo Y.Q., Huang M., Fang Y.H., Zou B.L. (2015). In situ synthesis and formation mechanism of ZrC and ZrB_2_ by combustion synthesis from the Co-Zr-B_4_C system. J. Asian Ceram. Soc..

[3] Zhang M.X., Zou B.L., Xu J.Y., Cai X.L., Wang Y., Huang M., Fang Y.H., Huo Y.Q., Cao X.Q. (2015). Reaction behavior, microstructure and application in coating of in situ ZrC-ZrB_2_ ceramic composites powders from a Co-Zr-B_4_C system. Mater. Des..

[4] Zhang M.X., Huo Y.Q., Hu Q.D., Zhang P., Zou B.L. (2014). Reaction behavior and formation mechanism of ZrC and ZrB_2_ in the Cu-Zr-B_4_C system. Int. J. Refract. Met. Hard Mater..

[5] Zhang M.X., Huo Y.Q., Huang M., Fang Y.H., Wang G.P. (2015). The effect of B_4_C particle size on the reaction process and product in the Cu-Zr-B_4_C system. J. Asian Ceram. Soc..

[6] Zhu G.L., Wang W., Wang R., Zhao C.B., Pan W.T., Huang H.J., Du D.F., Wang D.H., Shu D., Dong A.P. (2017). Formation mechanism of spherical TiC in Ni-Ti-C system during combustion synthesis. Materials.

[7] Liang Y.H., Wang H.Y., Yang Y.F., Du Y.L., Jiang Q.C. (2008). Reaction path of the synthesis of TiC-TiB_2_ in Cu-Ti-B_4_C system. Int. J. Refract. Met. Hard Mater..

[8] Zou B.L., Xu J.Y., Wang Y., Zhao S.M., Fan X.Z., Hui Y., Zhou X., Huang W.Z., Cai X.L., Tao S.Y. (2013). Self-propagating high-temperature synthesis of TiC-TiB_2_-based Co cermets from a Co-Ti-B_4_C system and fabrication of coatings using the cermet powders. Chem. Eng. J..

[9] Yang Y.F., Wang H.Y., Zhao R.Y., Liang Y.H., Jiang Q.C. (2008). Effect of Ni content on the reaction behaviors of self-propagating high-temperature synthesis in the Ni-Ti-B_4_C system. Int. J. Refract. Met. Hard Mater..

[10] Levashov E.A., Mukasyan A.S., Rogachev A.S., Shtansky D.V. (2017). Self-propagating high-temperature synthesis of advanced materials and coatings. Int. Mater. Rev..

[11] Jin S.B., Su H.K., Sha G. (2019). Atom Probe tomography analysis of TiC_x_ powders synthesized by SHS in Al/Fe/Cu-Ti-C systems. Materials.

[12] Matveev A.E., Promakhov V., Nikitin P., Babaev A., Vorozhtsov A. (2022). Effect of mechanical activation of Al-Ti-B powder mixture on phase composition and structure of Al-TiB_2_ composite materials obtained by self-propagating high-temperature synthesis (SHS). Materials.

[13] Xu J.Y., Zou B.L., Zhao S.M., Hui Y., Huang W.Z., Zhou X., Wang Y., Cai X.L., Cao X.Q. (2014). Fabrication and properties of ZrC-ZrB_2_/Ni cermet coatings on a magnesium alloy by atmospheric plasma spraying of SHS powders. Ceram. Int..

[14] Xu J.Y., Ma P.F., Zou B.L. (2021). Reaction mechanism of ZrB_2_-ZrC formation in Ni-Zr-B_4_C system analyzed by differential scanning calorimetry. Materials.

[15] Moore J.J., Feng H.J. (1995). Combustion synthesis of advanced materials: Part I: Reaction parameters. Prog. Mater. Sci..

[16] Barin I. (1995). Thermochemical Data of Pure Substances.

[17] Zou B.L., Shen P., Jiang Q.C. (2009). Dependence of the SHS reaction behavior and product on B_4_C particle size in Al-Ti-B_4_C and Al-TiO_2_-B_4_C systems. Mater. Res. Bull..

[18] Matveev A.E., Nikitin P.Y., Zhukov I.A., Zhukov A.S. (2021). The use of plastic waste as carbon raw materials to obtain TiC-based powders. Ceram. Int..

[19] Nikitin P.Y., Zhukov I.A., Matveev A.E., Sokolov S.D., Boldin M.S., Vorozhtsov A.B. (2020). AlMgB_14_-TiB_2_ composite materials obtained by self-propagating high-temperature synthesis and spark plasma sintering. Ceram. Int..

[20] Choi Y., Rhee S.W. (1993). Effect of aluminum addition on the combustion reaction of titanium and carbon to form TiC. J. Mater. Sci..

[21] Yang Y.F., Wang H.Y., Zhao R.Y., Jiang Q.C. (2009). Effect of reactant particle size on the self-propagating high-temperature synthesis reaction behaviors in the Ni-Ti-B_4_C system. Metall. Mater. Trans. A.

[22] Massalski T.B., Okamoto H., Subramanian P.R. (1990). Binary Alloy Phase Diagrams.

[23] Hayes E.T., Roberson A.H., Paasche O.G. (1953). The Zirconium-Nickel phase diagram. Trans. ASM.

